# Does sports participation affect the mediating role of impulsivity in the association between adverse childhood experiences and aggression?

**DOI:** 10.3389/fpsyg.2023.1234910

**Published:** 2024-01-08

**Authors:** Marija Janković, Geert Van Boxtel, Stefan Bogaerts

**Affiliations:** ^1^Department of Developmental Psychology, Tilburg University, Tilburg, Netherlands; ^2^Fivoor Science and Treatment Innovation (FARID), Rotterdam, Netherlands; ^3^Department of Cognitive Neuropsychology, Tilburg University, Tilburg, Netherlands

**Keywords:** reactive aggression, proactive aggression, adverse childhood experiences, sports participation, impulsivity

## Abstract

**Introduction:**

Adverse childhood experiences (ACEs) and self-reported aggression have often been associated, however, the mediating and moderating mechanisms underlying this association are not fully understood. In addition, less is known about potential factors that could serve as protection against the development of aggressive behavior. In this study, we investigated a moderated mediation model of sports participation and five impulsivity traits including positive and negative urgency, sensation seeking, and lack of premeditation and perseverance, in the association between ACEs and two forms of aggression.

**Methods and results:**

The sample comprised 651 (68.5% females) individuals from a Dutch community ranging in age from 18 to 82 years (*M* = 34.08). Contrary to our expectations, sports participation did not affect the associations of ACEs, the five impulsivity traits, and reactive and proactive aggression, except the association between ACEs and lack of premeditation. Specifically, the detrimental effect of ACEs on the propensity to act without thinking, increases among individuals with lower levels of sports participation. In addition, it was also found that sports participation directly contributed to higher levels of sensation seeking. Finally, after controlling for sociodemographic variables, the positive association between ACEs and reactive aggression was significantly mediated by negative urgency, lack of perseverance, and lack of premeditation, while the positive association between ACEs and proactive aggression was significantly mediated by lack of premeditation and positive urgency.

**Conclusion:**

The findings of this study add to the body of knowledge about the role of sports participation and impulsivity traits in the development of both forms of aggression, however, replication studies among multiple populations are needed before firm conclusions can be drawn.

## 1 Introduction

Aggression is disturbingly common in our society and disrupts public security (Madole et al., 2020). Aggressive behavior is any act intended to cause harm, pain, or injury to another individual (e.g., stabbing, shooting, or killing; Zirpoli, 2008; Allen and Anderson, 2017) and is often operationalized as reactive (i.e., impulsive, anger-oriented, and in response to a threat) and proactive aggression (i.e., goal-driven, intentional, and manipulative; Kempes et al., 2006; Bailey and Ostrov, 2008). A plethora of studies have attempted to explain the pathways to aggressive behavior by focusing on both distal and proximal influences. Recently, attention has also been paid to potential factors that could serve as protection against the development of aggressive behavior (Trajković et al., 2020; Zhu et al., 2022).

### 1.1 Distal and proximal risk factors related to aggression

In terms of distal factors, cross-sectional and longitudinal research linked adverse childhood experiences (ACEs; including maltreatment, physical/sexual abuse, poverty, and neglect) to various forms of aggressive behavior (e.g., Barnow and Freyberger, 2003; Côté et al., 2006; Kunst et al., 2010; Carver et al., 2014; Klein Tuente et al., 2020). According to the adaptive calibration model of stress responsivity (ACM; Ellis et al., 2017), individual differences in physiological responses to stress and provocation are largely determined by early experiences and environmental conditions and are thought to coordinate the development of slow (e.g., better self-regulation, tolerance to delayed gratification) versus fast (impulsivity, social antagonism, engagement in risky and aggressive behavior) life history strategies. In a biological sense, they operate in a way to maximize an individual’s likelihood of survival and reproduction and hence term adaptive. Activation of the stress response system during early childhood gives vital information on aspects of the child’s surroundings that are significant for life history strategies. Life history strategies have a tendency to shift toward the fast end of the life history continuum, characterized by increased risk-taking and impulsivity, when the activation of the stress response system is frequent and intense. This activation serves as a mechanism for conveying information about environmental unpredictability. On the other hand, in a secure environment, the stress response system is activated less frequently and at a low intensity. This, in turn, facilitates the development of slower life history strategies (e.g., growth and learning; Del Giudice et al., 2011). Based on the ACM model, four stress response patterns can be distinguished, namely sensitive, buffered, vigilant, and unemotional. Developmental exposure to high levels of stress can either upregulate (in the vigilant type) or downregulate (in the unemotional type) responsivity. Altered reactivity to stress, either in the direction of hyperreactivity or hyporeactivity, may indicate a disruption in the systems responsible for maintaining homeostasis. This may manifest in difficulties regulating emotions, thereby encouraging the pursuit of fast life history strategies (Ellis et al., 2017). Furthermore, research has shown that ACEs are more strongly associated with reactive aggression than proactive aggression (Brown et al., 2016, 2017; McRae et al., 2021). This is attributed to the belief that ACEs lead to dysregulation across affective (e.g., heightened levels of emotional reactivity), behavioral (e.g., poor impulse control), physiological (e.g., increased autonomic nervous system arousal), and cognitive (e.g., hostile attribution bias) patterns. This may, in turn, support the development of reactive aggression rather than proactive aggression because reactive aggression is affect-driven, while proactive aggression is goal-directed and predatory (Kempes et al., 2006; Bailey and Ostrov, 2008).

Regarding proximal factors, several studies showed that impulsivity is a direct risk for aggression as well as an important factor that serves as a pathway between ACEs and aggression (e.g., Madole et al., 2020; Jankovic et al., 2021; Meddeb et al., 2023). Impulsivity is a broad construct, and only a few studies distinguished between different forms of impulsivity, such as positive and negative urgency (emotion-related impulsivity), sensation seeking (excitement-seeking impulsivity), and lack of premeditation and perseverance (impulsivity related to deficits in conscientiousness; Cyders et al., 2014). Urgency represents a tendency to act hastily under circumstances of positive (positive urgency) or negative (negative urgency) affect. Sensation seeking refers to the need to seek out new and different sensations, feelings, and experiences. Lack of premeditation implies acting without thinking, while lack of perseverance refers to the inability to stay focused on a task (Cyders et al., 2014). Research has shown that emotion-related forms of impulsivity are more robustly linked to ACEs, internalizing and externalizing psychopathologies, as well as reactive and proactive aggression (Hecht and Latzman, 2015; Madole et al., 2020). For example, exposure to multiple ACEs was associated with negative urgency (Shin et al., 2018) and a recent meta-analysis found that emotion-related impulsivity traits (i.e., positive, and negative urgency) and lack of premeditation had a stronger association with aggression than other two scales (Bresin, 2019).

Furthermore, it has been shown that different impulsivity traits may be differentially associated with certain types of aggression. Hecht and Latzman (2015) found that reactive aggression was uniquely predicted by higher levels of negative urgency, followed by low perseverance, as well as high premeditation and low positive urgency. In contrast, proactive aggression was uniquely predicted by higher levels of positive urgency and, to a lesser extent, high premeditation. Derefinko et al. (2011) investigated how impulsivity traits are associated with self-reported interpersonal aggression and delinquent behavior in male undergraduates and found that a lack of premeditation and sensation seeking were relevant in predicting general violence, while urgency scales were more useful in predicting intimate partner violence. Mathias et al. (2007) also found that emotional distress was more strongly associated with impulsive aggression than premeditated aggression.

Taken together, previous findings suggest that different aspects of impulsivity may represent different mechanisms underlying the association between ACEs and specific subtypes of aggression. There is some evidence to suggest that emotional subtypes of impulsivity may better explain the link between ACEs and aggression in general (Madole et al., 2020). Similarly, urgency was an important mechanism linking emotional abuse with property crime and fraud, while lack of premeditation was a significant mediator in the association between child neglect and property crime (Shin et al., 2016). However, to the best of our knowledge, no previous research to date has investigated the possible mediating role of different impulsivity traits in the association between ACEs and two forms of aggression (i.e., reactive and proactive).

### 1.2 Sports participation as a protective factor against aggression

Moreover, it has been suggested that some activities, such as participation in sports, may improve personal resilience (i.e., the ability to bounce back from adversity) and lessen the detrimental effects of ACEs on various life outcomes in individuals who have suffered ACEs (Hughes et al., 2018; Norris and Norris, 2021). Sports participation can have a positive impact on physical and mental health in general and cardiovascular health and weight reduction in particular (Newman and Motta, 2007; Hughes et al., 2018). Sports participation has been shown to reduce depression and anxiety as well as to improve emotion regulation and coping skills (Newman and Motta, 2007; Panza et al., 2020). It has also been proven that moderate physical exercise improves the regulation of the autonomic nervous system, which exerts a significant impact on overall health (Daniela et al., 2022). In addition, engagement in team or group sports can boost social interaction and foster a sense of community (Newman and Motta, 2007; Sagatun et al., 2007). However, it has only recently become apparent that building resilience can be achieved through these factors that are enhanced during sports (e.g., friendship opportunities and access to role models), especially in individuals with ACEs. For example, a large Welsh study found that regular exercise reduced the prevalence of current mental illness across all ACE levels (Hughes et al., 2018). However, less is known about whether sports can also be seen as a beneficial strategy for reducing aggression, among other things.

Several studies documented that sports activity can reduce aggressive behavior (e.g., Trajković et al., 2020; Zhu et al., 2022). Participation in sports activities is assumed to deter aggressive behavior or delinquency, which is often referred to as the “deterrence” hypothesis (Schafer, 1969). This hypothesis assumes that youth who participate in organized sports are exposed to strong conformational influences, which lead them to internalize conventional norms and values rather than deviant ones (Hirschi, 1969; Schafer, 1969; Snyder, 1994). Therefore, focused sports interventions may be useful in individuals with ACEs and aggression problems to increase resilience and reduce aggression (Carver et al., 2014; Noel-London et al., 2021). Additionally, there is also the less researched “athletic delinquent” hypothesis, which claims that aggressive behavior is actually learned and stimulated by sports activities due to the possibility of cheating in athletic competitions (Begg et al., 1996). It has been shown that aggression is more common in young athletes because of highly competitive pressure and frustration (Oproiu, 2013). Although less studied, the delinquent hypothesis may find support in findings (e.g., Begg et al., 1996) documenting a positive link between sports participation and delinquent behavior. This hypothesis may be supported by a review showing that male youth of color living in urban communities and participating in competitive team contact sports were more likely to engage in aggressive and violent behavior (Newman et al., 2021). The aforementioned demonstrates that there is a lack of consistency in empirical findings regarding the impact of sports participation on aggression and therefore, more research is needed to understand whether sports participation may deter aggression or perhaps encourage it.

### 1.3 The present study

In summary, the association between ACEs and both forms of aggression is well established, with impulsivity playing an important role. However, the specific mediation of different impulsivity traits in the ACEs-aggression (reactive and proactive aggression) link remains unclear. In addition, sports participation has been proposed as a factor to improve emotion regulation, potentially reducing impulsivity and aggression, although conflicting evidence exists, showing that sports participation may increase frustration and aggression. Hence, further research is needed to elucidate the complex associations between ACEs, impulsivity traits, sports participation, and two forms of aggression.

**Figure 1:**
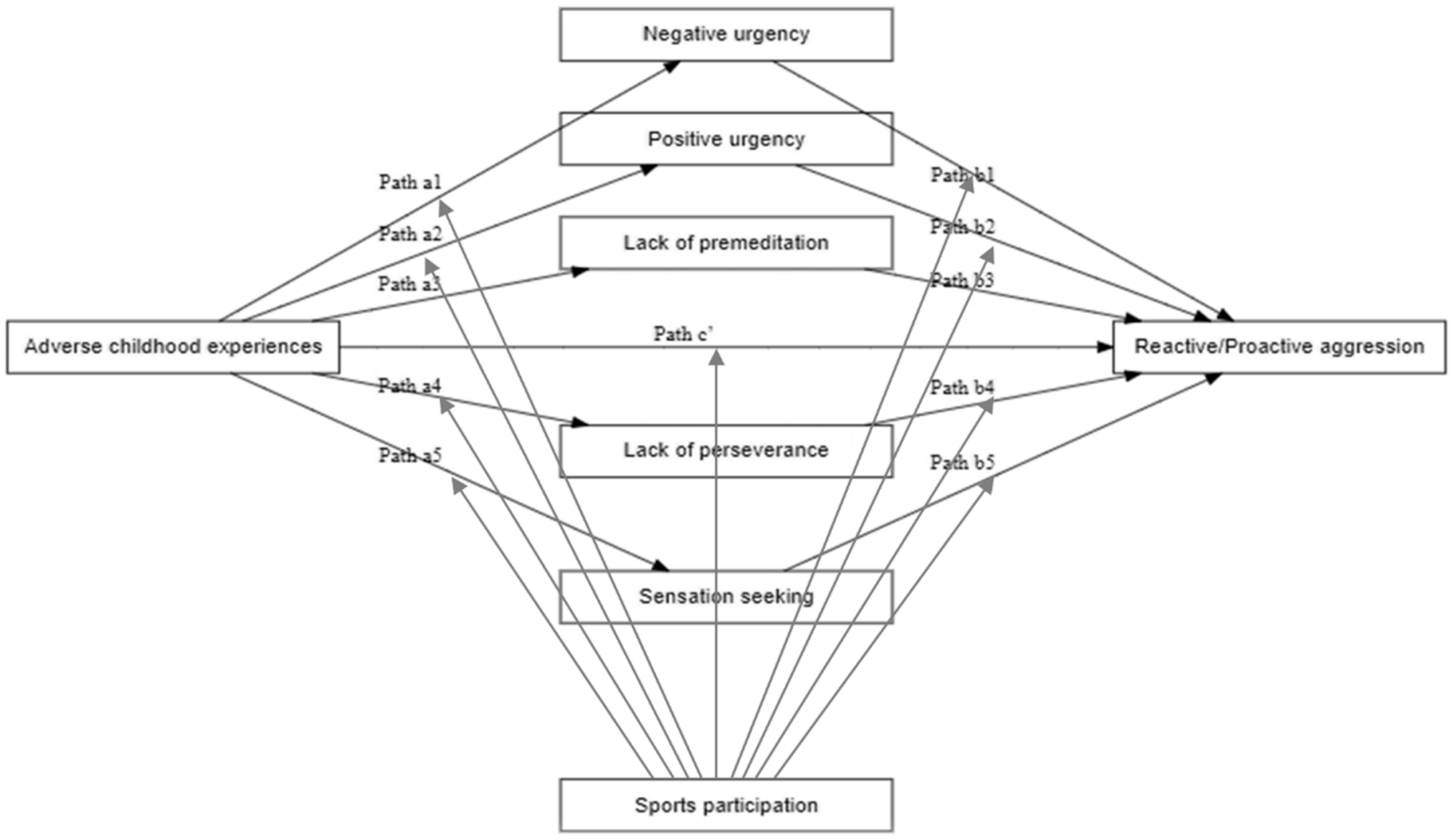
Conceptual model. ACEs, Adverse childhood experiences.

The aim of this study is therefore twofold. First, we investigated the extent to which the five different facets of impulsivity, including positive and negative urgency, sensation seeking, and lack of premeditation and perseverance, can mediate the association between ACEs and reactive and proactive aggression in a Dutch community sample. Second, we investigated the extent to which sports participation can moderate the association between ACEs and the five facets of impulsivity, the association between the five facets of impulsivity and reactive and proactive, as well as the association between ACEs and two forms of aggression. The conceptual model is presented in Figure 1. In all analyses, we controlled for the effect of sociodemographic variables (i.e., gender, age, ethnicity, and educational level). Based on the available evidence, the traits urgency and lack of premeditation were assumed to play a greater role in the association between ACEs and both forms of aggression than the other two impulsivity traits (sensation seeking and lack of perseverance), with negative urgency being more important for linking ACEs to reactive aggression and positive urgency being more important for linking ACEs to proactive aggression. Because of the mixed findings on the role of sports participation in the development of impulsivity and aggression, no specific hypothesis was formulated on the extent to which and how sports participation can influence these complex associations between ACEs, impulsivity traits, and aggression. Therefore, the second aim of our study should be considered rather exploratory.

## 2 Methods

### 2.1 Procedure and participants

The current study is part of an ongoing project aimed at investigating whether sports can promote resilience and general health in individuals with ACEs. Data collection lasted from March 2022 to January 2023. The survey was administered via the Qualtrics platform and participants were recruited by master’s students in clinical forensic psychology using a snowball sampling technique. Participants had to be at least 18 years or older and proficient in Dutch or English to be able to understand and complete the questionnaires. All participants were informed about the purpose of the study and consented to participate. The survey was completely anonymous and took approximately 30 min to complete. Participants received no compensation and could stop their participation at any time without giving a reason. The study was preregistered on OSF^1^ and approved by the Ethical Review Board of Tilburg University (reference number TSB_RP273).

The valid sample included 657 participants. Of them, 446 (67.9%) were females, 205 (31.2%) were males, 3 non-binary (0.5%) and 3 (0.5%) preferred not to report their gender. Non-binary participants and those who did not report their gender (*n* = 6) were excluded from the analysis. This resulted in a final sample of 651 participants (68.5% females), of which, 615 (94.5%) were Dutch-speaking participants and 36 (5.5%) were English-speaking participants. The age of the participants ranged from 18 to 82 years (*M* = 34.08, *SD* = 15.41). Most participants were Caucasian (*n* = 593, 91.1%) and single (*n* = 319, 47.6%), while others were in a relationship (*n* = 167, 25.7%) or married (*n* = 154, 23.7%). In general, participants were well-educated; 459 (70.5%) had a college or university degree, 60 (9.2%) had a graduate diploma, and 125 (19.2%) had a high-school diploma. At the time of the study, 258 (39.6%) were employed full-time and 157 (24.1%) part-time, 168 (25.8%) were students and 25 (3.8%) were retired. In the previous three months, on average, respondents participated in sports activities a few times a month (but not weekly). The most frequently practiced sports activities were indoor fitness/condition training (*n* = 188, 28.9%) followed by sport walking (*n* = 49, 7.5%), football (*n* = 46, 7.1%) and cycling (*n* = 34, 5.2%). In terms of gender differences, males were on average older, less educated and more likely to participate in the labor market than females. The complete overview of sample characteristics, including gender differences, can be found in Supplementary Table S1.

To establish the minimum sample size required for testing the study hypotheses, an *a priori* power analysis was performed using G*Power version 3.1.9.7. The analysis indicated that a sample size of *N* = 123 was needed for moderated mediation analysis, providing 80% power to detect a medium effect at a significance level of. 05. Therefore, the obtained sample size of *N* = 651 was sufficient to test the study’s hypotheses.

### 2.2 Measures

#### 2.2.1 ACEs

The Child Trauma Questionnaire-Short Form (CTQ-SF; Bernstein et al., 2003) was used to assess ACEs. The CTQ-SF is composed of 28 items, of which 25 measure three different forms of abuse including physical, sexual and emotional abuse, and two different forms of neglect including physical and emotional neglect, while the remaining three items are validity items. All items are rated on a 5-point Likert scale from 1 = *never true* to 5 = *very often true*. The composite ACE score was created by summing the scores across the 25 items where a higher total score indicated greater adversity. The internal consistency of the CTQ-SF in the present study was very good with Cronbach’s *α* = 0.87.

#### 2.2.2 Impulsivity

The short version of the S-UPPS-P Impulsive Behavior Scale (Cyders et al., 2014) was used to assess five facets of impulsivity (four items per dimension) namely negative urgency, lack of premeditation, lack of perseverance, sensation seeking, and positive urgency. The S-UPPS-P has 20 items rated on a 4-point Likert scale from 1 = *agree strongly* to 4 = *disagree strongly*. The scores of the five subscales were created by summing the scores on the respective items, with higher scores indicating higher levels of impulsivity. In the current sample, internal consistencies were: *α* = 0.58 (sensation seeking), *α* = 0.70 (negative urgency), *α* = 0.67 (lack of perseverance), *α* = 0.76 (positive urgency), and *α* = 0.78 (lack of premeditation). The internal consistency for sensation seeking was unacceptable (acceptable level of reliability ≤0.60; Hajjar, 2018). We, therefore, calculated a mean inter-item correlation (MIC), which is thought to be more appropriate for subscales with fewer items. MIC values between 0.15 and 0.50 suggest that items are fairly homogenous (Clark and Watson, 1995). We found acceptable MIC values for all subscales (sensation seeking = 0.27, lack of perseverance = 0.33, negative urgency = 0.37, positive urgency = 0.45, and lack of premeditation = 0.47) indicating that the items of the corresponding subscale represent the same content domain. Therefore, we retained all subscales in the current study.

#### 2.2.3 Aggression

The Reactive–Proactive aggression questionnaire (RPQ; Raine et al., 2006) was used to assess reactive aggression (11 items) and proactive aggression (12 items). All items are rated on a 3-point Likert scale from 0 = *never* to 2 = *often*. The reactive and proactive subscale scores were created by summing the scores on corresponding items. Higher scores indicated higher levels of aggression. In the current sample, internal consistency for reactive aggression was *α* = 0.81 indicating good reliability, while for proactive aggression, the internal consistency was acceptable with *α* = 0.74.

#### 2.2.4 Sports participation

To obtain data on sports participation, participants were asked whether and how often they participated in sports activities in the previous. The answer options were 1 = *never*, 2 = *once a year on average*, 3 = *once a month on average*, 4 = *a few times a month, but not weekly*, 5 = *once a week on average*, 6 = *twice a week on average*, 7 = *more than twice a week on average*, and 8 = *daily*. A higher score indicated more frequent sports participation. Additionally, participants were also asked to indicate which sport they played the most during that period by choosing one of the 14 categories offered (e.g., running, tennis, hockey) or by filling in the category other.

### 2.3 Statistical analysis

First, descriptive statistics including a sample distribution (i.e., skewness and kurtosis), correlations and gender differences were computed for the study variables using SPSS Statistics version 29. The z-scores were computed for all study variables to detect potential outliers. The z-scores larger than +3 or lower than −3 would be considered outliers and excluded from the analysis (Chikodili et al., 2021).

Second, the PROCESS macro extension (Hayes, 2017) was used to test mediation and moderation. In both the mediation and moderated mediation analyses, effects were estimated using linear models. These models were fitted utilizing a series of separate ordinary least squares regressions. Prior to estimating the linear models, the assumption of linearity was assessed by investigating residuals versus predicted value plots. All lines were approximately horizontal at zero, affirming that linearity assumption was met (see the Supplementary material for plots).

Subsequently, the PROCESS macro model 4 was applied to investigate whether the five different facets of impulsivity (i.e., negative urgency, premeditation, perseverance, sensation seeking, and positive urgency) mediated the associations between ACEs and two forms of aggression. The model was run separately for reactive and proactive aggression. Furthermore, the PROCESS macro model 59 was used to investigate the moderating effect of sports participation in the associations between ACEs and the five different facets of impulsivity (path a), between the five different facets of impulsivity and reactive and proactive aggression (path b) as well as between ACEs and two forms of aggression (path c’). Again, reactive and proactive aggression were investigated in separate models. In all analyses (i.e., model 4 and model 59), the continuous indicators were mean centered. Mean centering of predictor variables is recommended to enhance the interpretation of regression results when the predictor variables lack meaningful zero-points. It is also advised to mitigate nonessential multicollinearity when research questions include testing main effects alongside interactions (Cohen et al., 2014; Iacobucci et al., 2016). All analyses were also adjusted for gender, age, ethnicity, and educational level. Finally, we applied a bias-corrected bootstrap routine with 5,000 resamples to determine whether indirect effects in model 4 and model 59 were significant. The effect was significant if the bootstrap confidence intervals (CI) did not contain zero (Hayes, 2017).

## 3 Results

In the initial analysis, we first excluded non-binary participants and those who preferred not to report their gender (*n* = 6). Furthermore, within the remaining sample (*n* = 651), we identified 52 outliers (8.0%) using a z score threshold of ± 3 or more. In addition, based on skewness and kurtosis values (Field, 2009), the data violated the assumption of normality (see Supplementary Table S2). However, rather than excluding participants identified as outliers based on z scores, a decision was made to employ Tukey’s fences method with *k* = 3 (Tukey, 1977) for outlier removal. This is a more appropriate approach when data are not normally distributed, thereby avoiding potential bias resulting from the exclusion of a substantial number of participants (Kwak and Kim, 2017). Of note, the variables ACEs and proactive aggression violated the assumption of normality the most severely. To address this, we used the Box-Cox transformation (Box and Cox, 1964) to render ACEs approximately normally distributed. To transform proactive aggression, we applied a Square Root transformation due to the presence of scores of 0, rendering the Box-Cox transformation inappropriate in this case. Using Tukey’s fences method, no outliers were identified, and after transformation, both ACEs (skewness = 0.45 and kurtosis = −0.18) and proactive aggression approximated normal distribution (skewness = 0.62 and kurtosis = −0.51). Subsequently, we proceeded with descriptive analysis.

Questionnaire characteristics (i.e., means and standard deviations) including gender differences are presented in Table 1. Males scored significantly higher on positive urgency, sensation seeking, and lack of perseverance, as well as on both forms of aggression compared to females. In addition, correlations among study variables are presented in Table 2. Both reactive and proactive aggression exhibited significant positive correlations with ACEs, negative urgency, lack of premeditation, positive urgency, sensation seeking, male gender, and being white, while they displayed negative and significant correlations with education. Proactive aggression also showed a positive correlation with age. For a comprehensive overview of all correlations, see Table 2.

**Table 1:** Questionnaire characteristics.

Variable	Entire sample (*N* = 651)	Females (*n* = 446)	Males (*n* = 205)	Test statistics
	Mean (SD)			
ACEs	36.54 (9.56)	36.47 (9.82)	36.69 (8.99)	*F*(1,6,499) = 0.08
Impulsivity				
Negative urgency	9.35 (3.00)	9.44 (2.92)	9.17 (3.16)	*F*(1,649) = 1.11
Positive urgency	7.63 (3.16)	7.44 (3.10)	8.05 (3.25)	*F*(1, 649) = 5.38^*^
Lack of premeditation	7.06 (2.37)	7.06 (2.32)	7.04 (2.46)	*F*(1, 649) = 0.01
Lack of perseverance	7.29 (2.20)	7.15 (2.06)	7.59 (2.46)	*F*(1, 649) = 5.64^*^
Sensation seeking	10.54 (2.69)	10.06 (2.66)	11.59 (2.45)	*F*(1, 649) = 48.62^**^
Aggression				
Reactive aggression	6.25 (3.68)	5.70 (3.40)	7.45 (3.98)	*F*(1, 649) = 33.24^**^
Proactive aggression	1.44 (2.06)	0.98 (1.63)	2.44 (2.51)	*F*(1, 649) = 86.20^**^
Sport participation	4.30 (1.87)	4.27 (1.84)	4.35 (1.94)	*F*(1, 649) = 0.21

Test statistic refers to the test that was used to evaluate differences between males and females. ^*^p < 0.05, ^**^p < 0.01.

**Table 2:** Correlations among study variables.

Variable	1.	2.	3.	4.	5.	6.	7.	8.	9.	10.	11.	12.	13.
1. ACEs	-												
2. Negative urgency	0.21^**^	-											
3. Lack of premeditation	0.14^**^	0.32^**^	-										
4. Lack of perseverance	0.07	0.02	0.46^**^	-									
5. Positive urgency	0.19^**^	0.59^**^	0.39^**^	0.08^*^	-								
6. Sensation seeking	0.06	0.09^*^	0.08^*^	−0.01	0.25^**^	-							
7. Sports participation	−0.01	−0.04	−0.06	−0.01	−0.07	0.12^**^	-						
8. Reactive aggression	0.28^**^	0.26^**^	0.22^**^	−0.01	0.19^**^	0.14^**^	0.05	-					
9. Proactive aggression	0.27^**^	0.21^**^	0.23^**^	0.01	0.28^**^	0.14^**^	−0.04	0.58^**^	-				
10. Age	0.10^*^	−0.09^*^	−0.11^**^	−0.09^*^	−0.08^*^	−0.21^**^	−0.13^**^	−0.06	0.13^**^	-			
11. Gender^a^	−0.05	0.04	0.01	−0.09^*^	−0.09^*^	−0.26^**^	−0.02	−0.22^**^	−0.34^**^	−0.15^**^	-		
12. Education	−0.10^*^	−0.05	−0.06	0.06	−0.11^**^	0.09^*^	0.15^**^	−0.18^**^	−0.21^**^	−0.39^**^	0.17^**^	-	
13. Ethnicity^b^	0.16^**^	0.04	−0.01	−0.01	0.05	−0.01	0.02	0.11^**^	0.10^*^	−0.05	−0.01	−0.04	-

ACEs, Adverse childhood experiences. ^a^0 = male and 1 = female. ^b^0 = other and 1 = white. ^*^p < 0.05, ^**^p < 0.01.

To investigate whether five different facets of impulsivity, including positive and negative urgency, sensation seeking, and lack of premeditation and perseverance, could mediate the association between ACEs and reactive and proactive aggression, model 4 of the PROCESS macro was applied (Hayes, 2017). Reactive and proactive aggression were tested in separate models. Fit measures and overall model tests for linear regression models included in the mediation analysis with both reactive and proactive aggression are presented in Table 3. In the model with reactive aggression as the outcome variable, the results revealed that ACEs positively predicted reactive aggression (*b* = 0.10, *β* = 0.26, *t* = 7.18, *p* < 0.001) after controlling for gender, age, ethnicity, and educational level (i.e., sociodemographic variables). This means that individuals with higher levels of ACEs were more likely to display higher levels of reactive aggression. In addition, after controlling for sociodemographic variables, negative urgency (standardized indirect effect = 0.04, SE = 0.01, 95% CI [0.020, 0.066]), lack of perseverance (standardized indirect effect = −0.01, SE = 0.01, 95% CI [−0.025, −0.001]), and lack of premeditation (standardized indirect effect = 0.03, SE = 0.01, 95% CI [0.010, 0.052]) significantly mediated the positive association between ACEs and reactive aggression, while positive urgency and sensation seeking were not significant mediators in this association. Thus, higher levels of ACEs contributed to more negative urgency, lack of premeditation, and lack of perseverance, respectively. This, in turn, led to more reactive aggression when scoring higher on negative urgency or lack of premeditation, and less reactive aggression when scoring higher on lack of perseverance. The positive effect of ACEs on reactive aggression (*b* = 0.08, *β* = 0.22, *t* = 5.90, *p* < 0.001) remained significant after including impulsivity traits and sociodemographic variables in the model, which indicates partial mediation. For a complete overview of the results, see Table 4.

**Table 3:** Fit measures and overall model tests for linear regression models included in the mediation analysis.

Model	Dependent variable	R	R^2^	Overall model test			
				*F*	df1	df2	*p*
Mediator model							
	Negative urgency	0.257	0.066	9.093	5	644	< 0.001
	Positive urgency	0.265	0.070	9.699	5	644	< 0.001
	Lack of premeditation	0.214	0.046	6.213	5	644	< 0.001
	Lack of perseverance	0.165	0.272	3.604	5	644	0.003
	Sensation seeking	0.376	0.141	21.154	5	644	< 0.001
Full model							
	Reactive aggression	0.482	0.232	19.345	10	639	< 0.001
	Proactive aggression	0.523	0.274	24.070	10	639	< 0.001

Models estimated indirect, direct and total effects of adverse childhood experiences on reactive/proactive aggression, with negative urgency, positive urgency, lack of premeditation, lack of perseverance, and sensation seeking as mediating variables, and with gender, age, education and dichotomized ethnicity as covariates.

**Table 4:** Mediation model for reactive and proactive aggression.

Effect type	Effect	Estimate	SE	95% confidence intervals		*β*
				Lower	Upper	
Indirect effect	ACEs → Negative urgency → RA/PA	0.016/0.001	0.005/0.001	0.008/−0.001	0.027/0.003	0.048/0.002
	ACEs → Positive urgency → RA/PA	−0.006/0.002	0.004/0.001	−0.014/0.001	0.001/0.004	−0.014/0.002
	ACEs → Lack of premeditation → RA/PA	0.011/0.003	0.004/0.001	0.004/0.001	0.020/0.005	0.029/0.003
	ACEs → Lack of perseverance → RA/PA	−0.004/−0.001	0.002/0.001	−0.001/−0.002	−0.004/0.001	−0.011/−0.001
	ACEs → Sensation seeking → RA/PA	0.001/0.001	0.001/0.001	−0.001/−0.001	0.004/0.001	0.003/0.001
Path a	ACEs → Negative urgency	0.070	0.012	0.046	0.095	0.221
	ACEs → Positive urgency	0.062	0.013	0.036	0.087	0.184
	ACEs → Lack of premeditation	0.039	0.010	0.020	0.058	0.154
	ACEs → Lack of perseverance	0.019	0.009	0.001	0.038	0.083
	ACEs → Sensation seeking	0.021	0.011	0.001	0.042	0.073
Path b	Negative urgency → RA/PA	0.229/0.020	0.054/0.124	0.123/−0.004	0.334/0.044	0.187/0.069
	Positive urgency → RA/PA	−0.091/0.027	0.054/0.012	−0.197/0.002	0.015/0.051	−0.781/0.097
	Lack of premeditation → RA/PA	0.292/0.073	0.068/0.016	0.159/0.042	0.424/0.103	−0.188/0.198
	Lack of perseverance → RA/PA	−0.216/−0.047	0.067/0.015	−0.348/−0.077	−0.084/−0.016	−0.130/−0.118
	Sensation seeking → RA/PA	0.053/0.009	0.053/0.012	−0.051/−0.001	0.156/0.033	0.039/0.028
Direct effect	ACEs → RA/PA	0.084/0.016	0.014/0.003	0.056/0.009	0.112/0.022	0.215/0.172
Total effect	ACEs → RA/PA	0.103/0.021	0.014/0.003	0.075/0.015	0.131/0.028	0.263/0.227
Covariates	Gender → Negative urgency	0.304	0.251	−0.188	0.796	0.047
	Gender → Positive urgency	−0.547	0.263	−1.091	−0.057	−0.085
	Gender → Lack of premeditation	0.031	0.200	−0.361	0.423	0.006
	Gender → Lack of perseverance	−0.520	0.188	−0.889	−0.151	−0.110
	Gender → Sensation seeking	1.764	0.215	−2.186	−1.341	−0.305
	Gender → RA/PA	−1.591/−0.569	0.290/0.067	−2.160/−0.700	−1.022/−0.437	−0.201/−0.305
	Age → Negative urgency	−0.027	0.008	−0.043	−0.011	−0.140
	Age → Positive urgency	−0.034	0.009	−0.051	−0.018	−0.167
	Age → Lack of premeditation	−0.025	0.007	−0.038	−0.012	−0.164
	Age → Lack of perseverance	0.019	0.009	0.001	0.038	−0.099
	Age → Sensation seeking	−0.044	0.007	0.001	0.042	−0.249
	Age → RA/PA	−0.044/0.001	0.009/0.002	0.075/−0.004	0.131/0.005	−0.185/0.014
	Ethnicity → Negative urgency	−0.093	0.408	−0.893	0.707	−0.009
	Ethnicity → Positive urgency	0.033	0.428	−0.808	0.873	0.003
	Ethnicity → Lack of premeditation	−0.377	0.325	−1.015	0.260	−0.046
	Ethnicity → Lack of perseverance	−0.188	−0.305	−0.788	0.411	−0.024
	Ethnicity → Sensation seeking	−0.302	0.350	−0.990	0.386	−0.032
	Ethnicity → RA/PA	0.591/0.165	0.471/0.109	−0.334/−0.049	1.515/0.379	0.046/0.054
	Education → Negative urgency	−0.490	0.217	−0.9166	−0.064	−0.094
	Education → Positive urgency	−0.745	0.228	−1.193	−0.297	−0.136
	Education → Lack of premeditation	−0.456	0.173	−0.796	−0.796	−0.112
	Education → Lack of perseverance	0.156	0.163	−0.163	0.476	0.041
	Education → Sensation seeking	0.209	0.187	−0.158	0.575	0.045
	Education → RA/PA	−1.229/−0.2004	0.251/0.058	−1.722/−0.314	−0.736/−0.087	−0.193/−0.134

ACEs, Adverse childhood experiences; RA, Reactive aggression; PA, Proactive aggression; Ethnicity (0 = other and 1 = white); Gender (0 = male and 1 = female).

In the model with proactive aggression as the outcome variable, results showed that ACEs positively predicted proactive aggression (*b* = 0.02, *β* = 0.22, *t* = 6.32, *p* < 0.001) after controlling for sociodemographic variables. Individuals with higher levels of ACEs more often exhibited higher levels of proactive aggression. In addition, after controlling for sociodemographic variables, only positive urgency (standardized indirect effect = 0.02, SE = 0.01, 95% CI [0.002, 0.037]) and lack of premeditation (standardized indirect effect = 0.03, SE = 0.01, 95% CI [0.012, 0.054]) significantly mediated the positive association between ACEs and proactive aggression, while the other three impulsivity traits were not significant mediators in this association. Thus, higher levels of ACEs led to more lack of premeditation and more positive urgency respectively, which in turn contributed to higher levels of proactive aggression. The positive effect of ACEs on proactive aggression (*b* = 0.02, *β* = 0.17, *t* = 4.83, *p* < 0.001) remained significant after including impulsivity traits and sociodemographic variables in the model, which indicates partial mediation. For a complete overview of the results, see Table 4.

**Figure 2:**
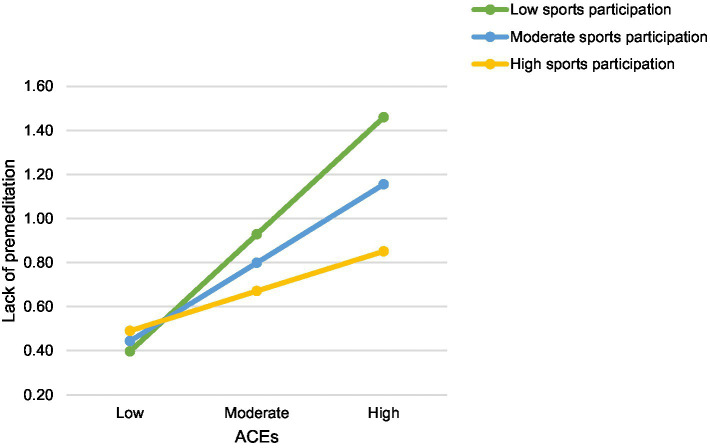
Moderation of sports participation. ACEs, Adverse childhood experiences.

Model 59 of the PROCESS macro was used (Hayes, 2017) to investigate a moderated mediation of sports participation and five impulsivity traits in the association between ACEs and both forms of aggression (i.e., reactive and proactive). Reactive and proactive aggression were tested in separate models. In the model with reactive aggression as the outcome variable, we found no evidence for a moderated mediation. Sports participation was a significant moderator only in the association between ACEs and the lack of premeditation, *R*^2^ change = 0.06, *F*(1, 642) = 4.18, *p* = 0.04. Simple slope analysis further revealed that the effect of ACEs on lack of premeditation was significant at low level (*b* = 0.06, *SE* = 0.01, *t* = 4.34, 95% CI [0.031, 0.082]) and mean level (*b* = 0.04, *SE* = 0.01, *t* = 3.83, 95% CI [0.018, 0.057]) of the moderator, but not at high level (*b* = 0.02, *SE* = 0.01, *t* = 1.39, 95% CI [−0.008, 0.046]). This indicates that as levels of sports participation decrease, the positive effect of ACEs on lack of premeditation becomes stronger. Figure 2 depicts a graphical presentation of the simple slope analysis. Moreover, sports participation did not exert a moderating effect on the association between ACEs and the other four impulsivity traits. Likewise, it did not moderate the association of five impulsivity traits and ACEs with reactive aggression. However, sports participation had a significant positive direct effect on sensation seeking (*b* = 0.11, *t* = 2.03, *p* = 0.04), but not on other variables in the model. This indicated that individuals who participated more frequently in sports activities were more likely to display higher levels of sensation seeking. A data script of this analysis is available in the Supplementary material.

Likewise, in the model with proactive aggression as the outcome variable, there was no evidence of moderated mediation. Sports participation did not significantly moderate the association between ACEs and any of the five impulsivity traits, or the association of five impulsivity traits and ACEs with proactive aggression. As in the analysis with reactive aggression, this model also revealed the direct positive effect of sports participation on sensation seeking (*b* = 0.11, *t* = 2.03, *p* = 0.04). There were no other significant direct effects of sports participation on remaining impulsivity traits or proactive aggression. The data analysis script is provided in the Supplementary material.

## 4 Discussion

ACEs and self-reported aggression are often associated, however, the mediating and moderating factors that may underlie this association are not yet fully understood. In this study, we investigated a moderated mediation model of sports participation and five impulsivity traits in the association between ACEs and reactive and proactive aggression in individuals from a Dutch community. The findings of this study partially support our hypothesis and bring new insights into the role of ACEs, impulsivity traits and sports participation in the development of reactive and proactive aggression.

Consistent with the literature, we found that individuals with more ACEs were more likely to display higher levels of reactive and proactive aggression, respectively (Barnow and Freyberger, 2003; Côté et al., 2006; Carver et al., 2014). ACEs are thought to foster the development of fast life history strategies, such as impulsivity, social antagonism, and engagement in risky and aggressive behavior (Ellis et al., 2017) as well as to cause impaired emotion regulation (Schmitz et al., 2021). It came as no surprise that the effect of ACEs was stronger for reactive aggression than for proactive aggression. This could be explained by the fact that reactive aggression is characterized by emotional lability and is therefore often reflected in impulsive reactions after provocation, while proactive aggression is driven by low emotionality and high levels of instrumentality that serve as a means of obtaining benefits (Romero-Martínez et al., 2022).

Furthermore, our hypothesis that urgency traits and lack of premeditation would have a greater role in the association between ACEs and both forms of aggression than the other two impulsivity traits (sensation seeking and lack of perseverance) found some support in the findings of the present study. Specifically, in line with our hypothesis, we found that negative urgency and lack of premeditation were significant mediators in the association between ACEs and reactive aggression, with the latter being also a significant mediator in the association between ACEs and proactive aggression along with positive urgency. This means that individuals who experienced more ACEs were more likely to act rashly when upset or to make decisions without considering their consequences. This in turn was associated with more reactive aggression and more proactive aggression in the case of not thinking or planning before acting. In addition, our findings showed that higher levels of proactive aggression in individuals with an increasing number of ACEs can be partially attributed to their propensity for impulsive responses when experiencing positive affect. Our findings are consistent with previous findings showing that urgency scale and lack of premeditation are important factors linking ACEs and aggression (Shin et al., 2016; Madole et al., 2020). Recently, it has been suggested that positive and negative urgency should rather be considered as one construct, as the same cognitive and affective pathways are involved in urgency-related behaviors (Billieux et al., 2021). Our study found that urgency scales played different roles in the association between ACEs and two forms of aggression. Thus, our results generally support the findings of Cyders and Smith (2008), emphasizing the need to distinguish between positive and negative urgency.

Moreover, we found that lack of perseverance was a significant mediator in the association between ACEs and reactive aggression. That is, individuals with more ACEs were less likely to remain focused on a task, but surprisingly this was linked to less reactive aggression. Although previous research demonstrated that low perseverance led to higher levels of reactive aggression (Hecht and Latzman, 2015), our study showed the opposite. This discrepancy in results may be attributed to the characteristics of the sample. Hecht and Latzman (2015) focused on undergraduate students, whereas a more diverse sample from the community was included in our study. It is reasonable to believe that students are more goal-oriented, especially when it comes to achieving their academic goals, and that failing to stay focused on a task can lead to unpleasant emotions such as frustration and anger, which then may fuel more reactive aggression. Conversely, less perseverance in a more diverse community sample is likely to just result in easier distraction rather than necessarily more aggressive behavior. Finally, we expected that sensation seeking (together with lack of perseverance) would play a smaller role in the relationship between ACEs and aggression than the other impulsivity traits, but it turned out that sensation seeking had no effect at all on this relationship. Nevertheless, this finding is in line with a study that found that sensation seeking was not a significant mediator in the association between ACEs and different types of crime (e.g., property crime, violent crime, and fraud; Shin et al., 2016).

Regarding the exploratory part of our study on the extent to which sports participation may influence the complex associations between ACEs, impulsivity traits, and aggression, no evidence for a moderated mediating effect of sports participation and impulsivity traits on the association between ACEs and aggression was found. In addition, sports participation did not moderate the associations of the five impulsivity traits and ACEs with both forms of aggression. However, it did act as a moderator in the association between ACEs and lack of premeditation. This moderation revealed that ACEs have a larger adverse effect on an individual’s tendency to act without considering the potential consequences when their participation in sports declines. In other words, this finding suggests that sports involvement may function as a protective factor for individuals with ACEs, helping mitigate the development of the impulsivity trait known as a lack of premeditation. This aligns with prior research that highlighted the role of sports engagement in fostering resilience (White and Bennie, 2015; Norris and Norris, 2021). Through sports, individuals with ACEs can enhance their social skills, and learn to seek support from others rather than succumbing to impulsive behavior. Engaging in sports can also boost cognitive functionality, enabling them to carefully evaluate the likely outcomes of their actions before proceeding, ultimately assisting them in reaching their goals.

Furthermore, our study revealed that sports participation had a positive direct effect on sensation seeking. This means that individuals who play sports more frequently are more likely to develop a tendency to seek new and different sensations, feelings, and experiences. Research consistently found that high-sensation seekers engage in more (risky) sports than low-sensation seekers (Zuckerman, 1983; Rowland et al., 1986; McEwan et al., 2019). It is plausible that this association is bidirectional. On the one hand, high-sensation seekers tend to take physical risks to achieve new, varied, and intense experiences and sample a wide selection of activities. In addition, seeking an optimal level of stimulation can also be seen as a good fit for participants in high-risk sports (Zuckerman, 1983). On the other hand, physical activity causes the release of endorphins and dopamine in the nervous system and when the activity is completed, the release of these chemicals is stopped (Leuenberger, 2006). To trigger the release of endorphins and dopamine, which are thought to create a feeling of pleasure or reward, one may be more driven to intensify their participation in a sport, which can even result in a sports addiction (Leuenberger, 2006). The same chemical processes are thought to motivate people to seek new and exciting experiences (Norbury and Husain, 2015). Thus, it may be argued that the more physical activity a person engages in, the more reliant they may become on endorphins and dopamine, and as a result, develop a need for a thrill-seeking experience to boost levels of these chemicals.

Furthermore, our findings do not favor either the so-called athletic delinquent hypothesis, which indicates that aggressive behavior is learned and stimulated by sports activity (Begg et al., 1996; Oproiu, 2013; Newman et al., 2021) or the “deterrence” hypothesis (Schafer, 1969), which assumes that sports activity can reduce aggressive behavior (e.g., Trajković et al., 2020; Zhu et al., 2022). We found no direct effect of sports participation on reactive and proactive aggression, respectively. Previous studies explained the co-occurrence of involvement in sports and (reactive) aggressive behavior, for example, through Bandura’s social learning theory (Cusimano et al., 2016), frustration caused by failure to win (Nucci and Young-Shim, 2005), or sports context (e.g., sport settings and sport types; Kreager, 2007). In the context of social learning theory, it has been argued that a “group can play a significant role in shaping players’ attitudes and behaviors through observation and modeling” (Cusimano et al., 2016, p. 3). Bortoli et al. (2012) further revealed that the moral climate of the team, created by both coaches and teammates, was a crucial determinant of aggressive acts. Last but not least, engagement in non-contact sports (e.g., tennis) reduced aggressive and violent behavior, while engagement in contact sports (e.g., football) encouraged physically aggressive and violent behavior (Kreager, 2007). In our study, we grouped all sports into a single category without considering various sports classifications or contexts, which may have led to disparities between our results and earlier research findings. As suggested earlier, this could significantly influence the nature of the relationship between sports participation and aggression.

Finally, our study adds to the literature that sports participation does not affect the associations between ACEs, impulsivity traits, and two forms of aggression, except for the association between ACEs and lack of premeditation. This area of research is still in its infancy and more empirical studies are needed to draw firmer conclusions. For example, a large nationwide Welsh study found that regular sports participation in childhood was associated with lower levels of mental illness at all ACE levels (Hughes et al., 2018), suggesting that sports participation is an effective intervention for improving the mental health of those who have been victimized. However, based on findings from our study, the same is not true when it comes to managing aggression in individuals with ACEs; yet sports participation can help these individuals with ACEs become less impulsive and behave without thinking. It is important to note that our study did not consider contextual factors such as individual versus team sport, or non-contact versus contact sport. It is still possible that certain sports could be beneficial in reducing aggression. In addition, the individuals in our sample had on average low levels of ACEs (Bernstein et al., 2003), and therefore, sports participation as an aggression inhibitor may be different among more traumatized individuals. However, our study revealed that in individuals with ACEs, treating both negative urgency and lack of premeditation could have promising effects on deterring reactive aggression, whereas treating lack of premeditation and positive urgency might have promising effects on deterring proactive aggression. Before drawing any firm conclusion based on our findings, the limitations of this study should be acknowledged.

The first limitation concerns the use of a snowball sampling method to recruit participants from the community, which complicates the generalizability of our findings. A replication study on a more representative sample of Dutch community individuals is recommended. It would also be interesting to conduct this study in a sample of forensic psychiatric patients as they are characterized by more ACEs and aggressive behavior (Jankovic et al., 2021). Another limitation is that all sports were placed under a single category, even though certain sports, such as group sports or contact sports may have differing influences on the emergence of aggressive behavior (Kreager, 2007; Oproiu, 2013). Furthermore, in our study, particular impulsivity scales such as lack of perseverance and sensation seeking showed low reliability, which could be attributed to the small number of items that comprised each scale. Nonetheless, Cronbach’s alpha tends to underestimate the internal consistency and is sensitive to the number of items in the scale (Nunnally, 1994). Therefore, it is recommended to rely on composite scores, which in this study showed good reliability for the S-UPPS-P instrument (*α* = 0.78 for). Finally, we tested reactive and proactive aggression in separate models. Since these two forms of aggression shared around 30% of the variance in our study, more comprehensive models that include both forms of aggression in a single analysis may be explored in future research. This could help reveal the unique associations between two types of aggression and the variables of interest.

To conclude, the societal and economic costs of aggression are very high (Foster and Jones, 2005) and it is thus necessary to better understand the factors that contribute to aggression. In this study, we found that the association between ACEs and the two forms of aggression was significantly mediated by lack of premeditation. In addition, the association between ACEs and reactive aggression was partially explained by negative urgency and lack of perseverance, while the association between ACEs and proactive aggression was partially explained by positive urgency. With the exception of the association between ACEs and lack of premeditation, sports participation did not appear to affect the complex associations between ACEs, the five impulsivity traits, and two forms of aggression. Due to the paucity of evidence on the topic under investigation and the limitations that were present in this study, replication studies among multiple target groups are needed before firm conclusions can be drawn.

## Data availability statement

The raw data supporting the conclusions of this article will be made available by the authors, without undue reservation.

## Ethics statement

The studies involving humans were approved by the Ethical Review Board of Tilburg University (reference number TSB_RP273). The studies were conducted in accordance with the local legislation and institutional requirements. The participants provided their written informed consent to participate in this study.

## Author contributions

MJ analyzed the data and wrote the first draft of the manuscript. SB and GB critically revised the manuscript for important intellectual content. All authors contributed to and have approved the final manuscript.

## Footnotes

^1^ https://osf.io/e9sbr/
