# Supplementary material for: Does sports participation affect the mediating role of impulsivity in the association between adverse childhood experiences and aggression?
Source: Front Psychol. 2024 Jan 8;14:1234910. doi: 10.3389/fpsyg.2023.1234910 (PMC10800594; doi:10.3389/fpsyg.2023.1234910)
Supplement: Supplementary file 1 [file Data_Sheet_1.docx]

Supplementary Material

Does Sports Participation Affect the Mediating Role of Impulsivity in the Association Between Adverse Childhood Experiences and Aggression?

Marija Janković^*^, Geert Van Boxtel, Stefan Bogaerts

*** Correspondence:** Marija Janković: M.Jankovic_1@tilburguniversity.edu

# Supplementary Tables

Table S1

Sample Characteristics

|  | Entire sample  (*N*=651) | Females  (*n*=446) | Males (*n*=205) | Test statistics |
| --- | --- | --- | --- | --- |
| Variable | *Mean* (*SD*)/*N* (%) | | |  |
| Age | 34.08 (15.41) | 32.52 (14.36) | 37.50 (17.03) | *F*(1,649)=14.99^**^ |
| Dutch-speaking (vs English-speaking) participants | 615 (94.5%) | 424 (95.1%) | 191 (93.2%) | χ^2^(1)=0.97 |
| Ethnicity |  |  |  |  |
| White/Caucasian | 593 (91.1%) | 407 (91.3%) | 186 (90.7%) | χ^2^(5)=7.39 |
| Hispanic/Latino | 3 (0.5%) | 2 (0.4%) | 1 (0.5%) |  |
| Black/African American | 5 (0.8%) | 1 (0.2%) | 4 (2.0%) |  |
| Asian | 12 (1.8%) | 10 (2.2%) | 2 (1.0%) |  |
| Prefer not to say | 4 (0.6%) | 2 (0.4%) | 2 (1.0%) |  |
| Other | 34 (5.2%) | 24 (5.4%) | 10 (4.9%) |  |
| Education |  |  |  | χ^2^(4)=20.06^***^ |
| No formal education | 3 (0.5%) | 1 (0.2%) | 2 (1.0%) |  |
| Primary | 4 (0.6%) | 2 (0.4 %) | 2 (1.0%) |  |
| High school | 125 (19.2%) | 67 (15.0%) | 58 (28.3%) |  |
| College/University | 459 (70.5%) | 329 (73.8%) | 130 (63.4%) |  |
| Graduate school | 60 (9.2%) | 47 (10.5%) | 13 (6.3%) |  |
| Marital status |  |  |  | χ^2^(4)=8.15 |
| Single | 310 (47.6%) | 229 (51.3%) | 81 (39.5%) |  |
| In a relationship | 167 (25.7%) | 107 (24.0%) | 60 (29.3%) |  |
| Married | 154 (23.7%) | 97 (21.7%) | 57 (27.8%) |  |
| Divorced | 13 (2.0%) | 8 (1.8%) | 5 (2.4%) |  |
| Widowed | 7 (1.1%) | 5 (1.1%) | 2 (1.0%) |  |
| Employment |  |  |  | χ^2^(6)=62.56^***^ |
| Full time employment | 258 (39.6%) | 139 (31.2%) | 119 (58.0%) |  |
| Part time employment | 157 (24.1%) | 134 (30.0%) | 23 (11.2%) |  |
| Unemployed/Looking for work | 6 (0.9%) | 5 (1.1%) | 1 (0.5%) |  |
| Unemployed/Not looking for work | 4 (0.6%) | 4 (0.9%) | 0 (0.0%) |  |
| Student | 168 (25.8%) | 122 (27.4%) | 46 (22.4%) |  |
| Retired | 25 (3.8 %) | 12 (2.7%) | 13 (6.3%) |  |
| Other | 33 (5.1%) | 30 (6.7%) | 3 (1.5%) |  |

*Note*. Test statistic refers to the test that was used to evaluate differences between males and females.

^***^ p < .001.

Table S2

Skewness and Kurtosis of Study Variables (*N* = 651)

| Variable | Skewness | Kurtosis |
| --- | --- | --- |
| Age | 1.10 | - 0.34 |
| Sport participation | - .54 | - .86 |
| ACEs | 2.50 | 9.25 |
| Positive urgency | .41 | - 1.01 |
| Negative urgency | - .32 | - .78 |
| Sensation seeking | - .33 | - .28 |
| Lack of perseverance | .72 | .78 |
| Lack of premeditation | .72 | .24 |
| Reactive aggression | .71 | .47 |
| Proactive aggression | 2.19 | 5.69 |

*Note*. ACEs = Adverse childhood experiences.

# Assumption of Linearity

## Outcome Variable: Reactive Aggression

| 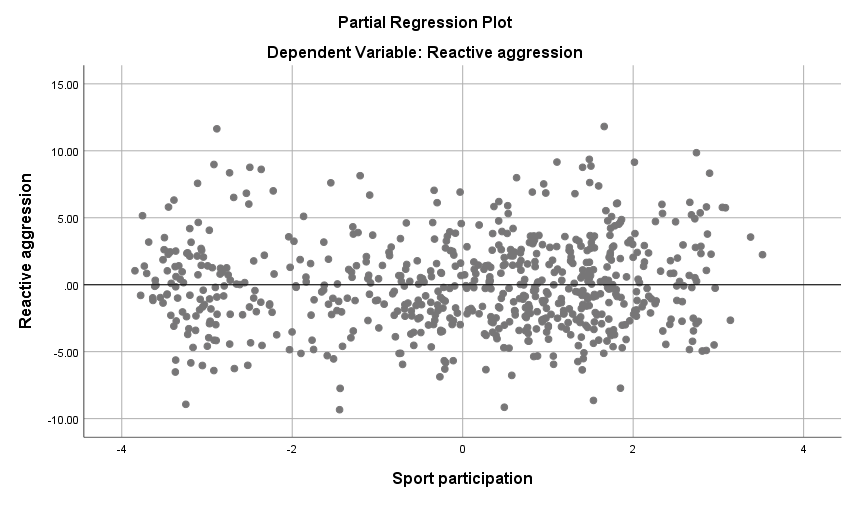 | 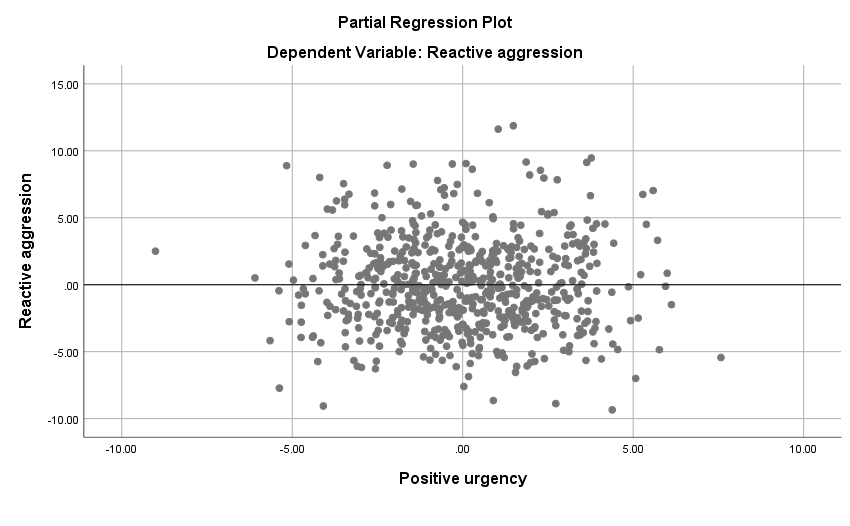 |
| --- | --- |
| 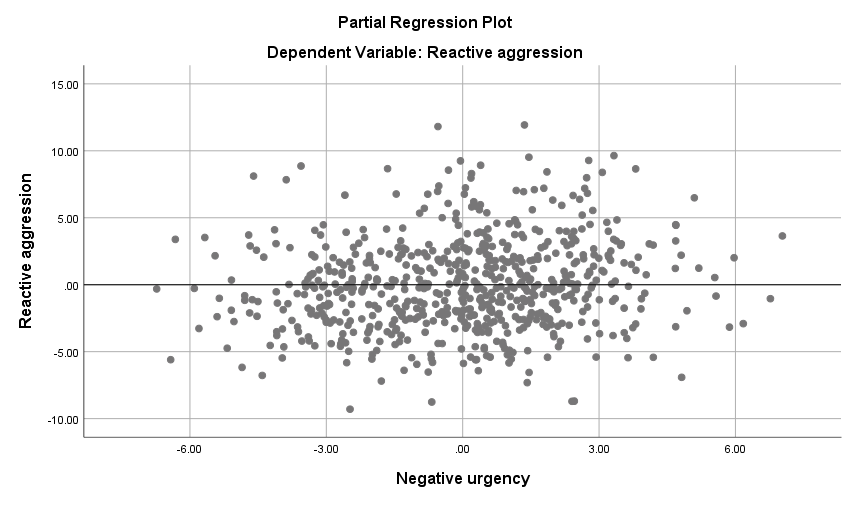 | 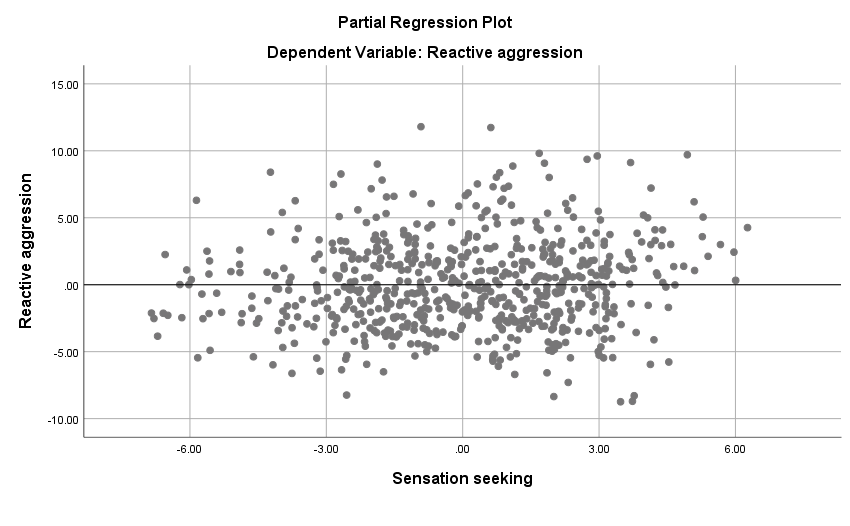 |
| 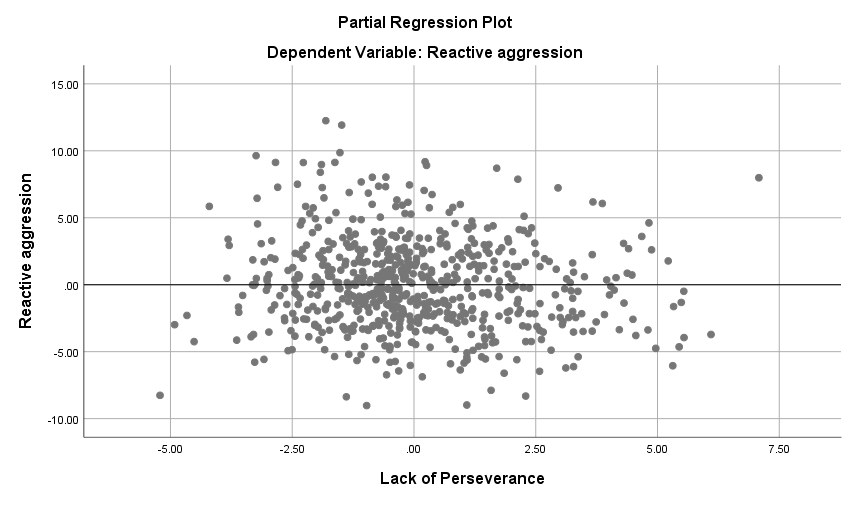 | 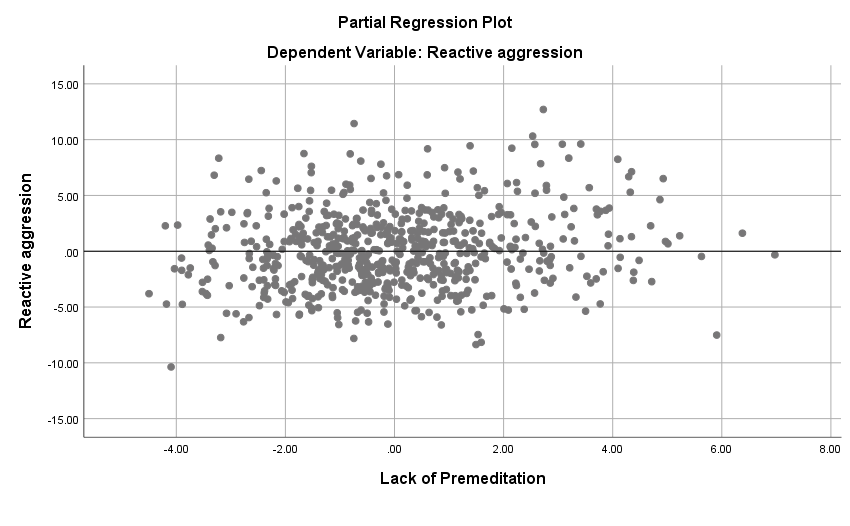 |
| 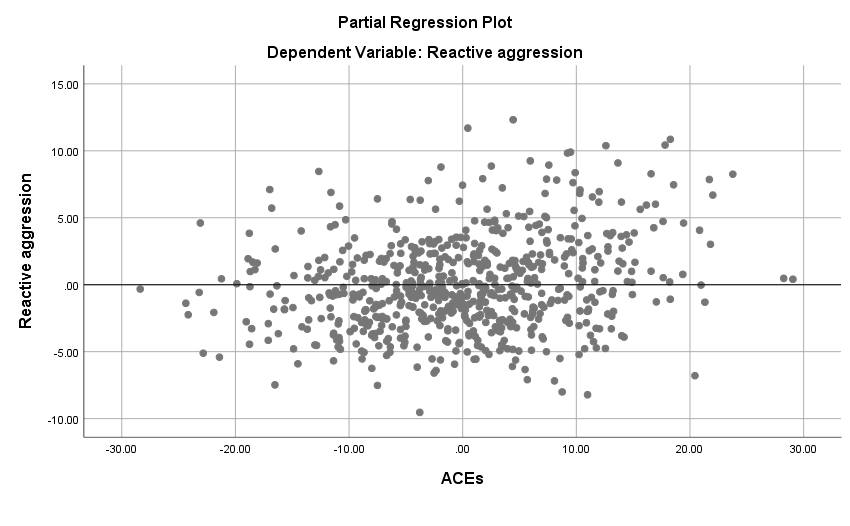 |  |

## Outcome Variable: Reactive Aggression

| 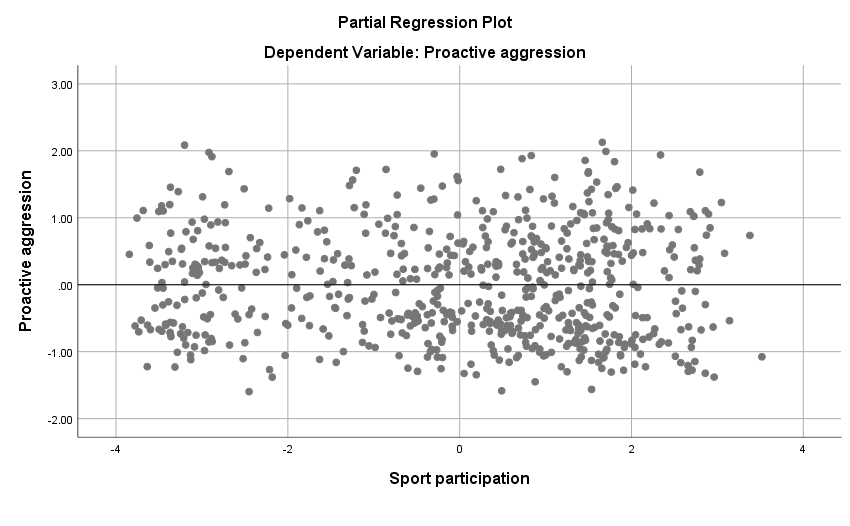 | 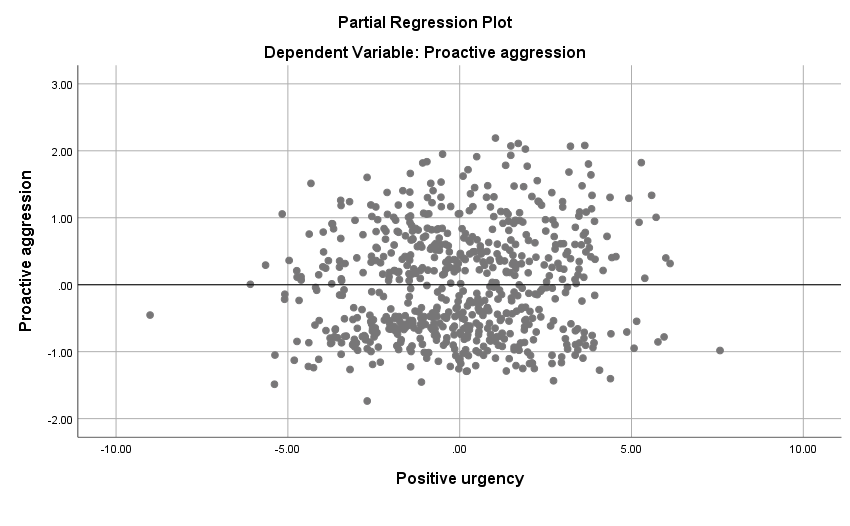 |
| --- | --- |
| 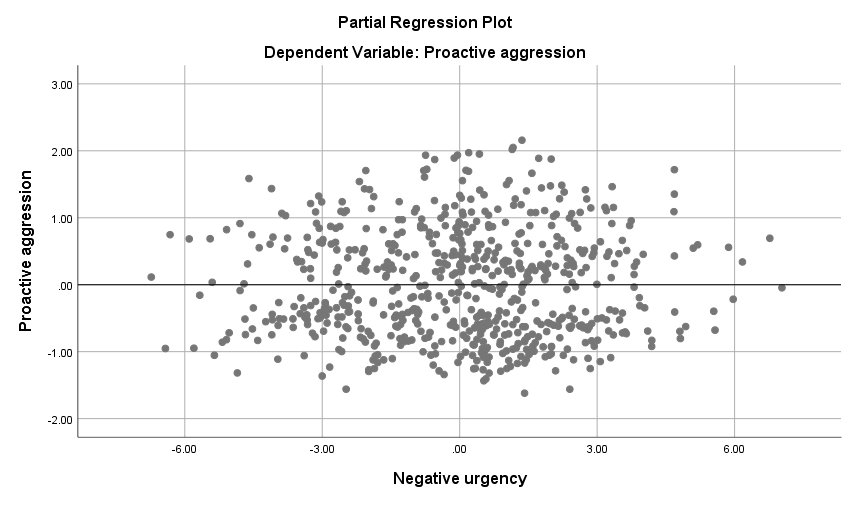 | 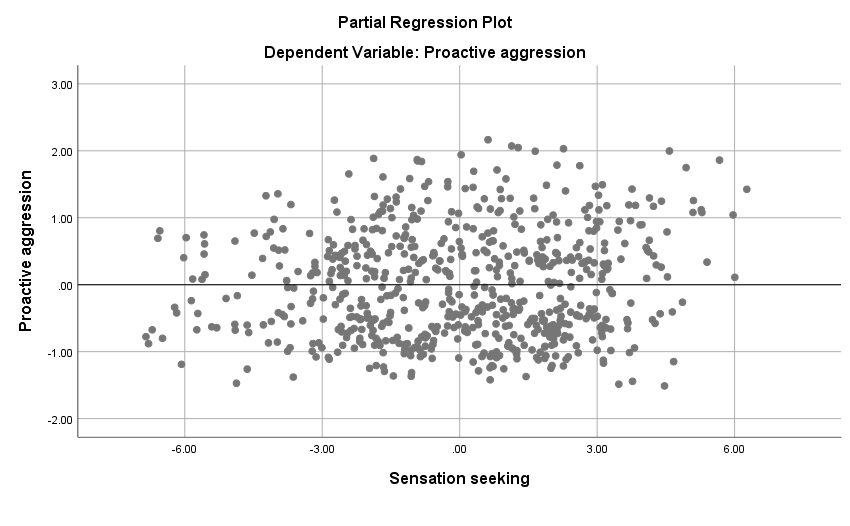 |
| 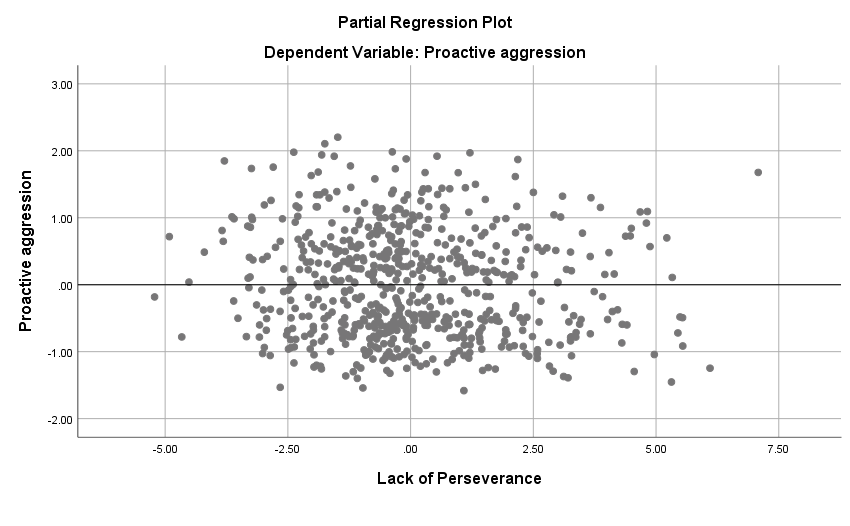 | 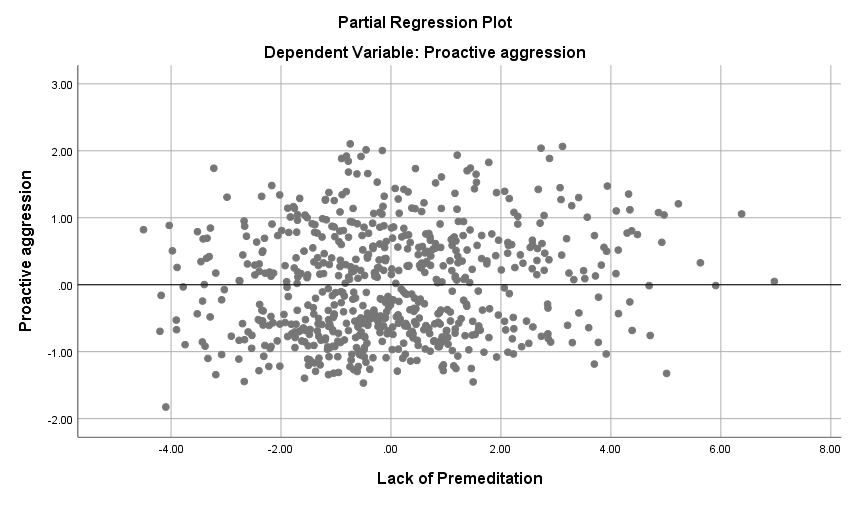 |
| 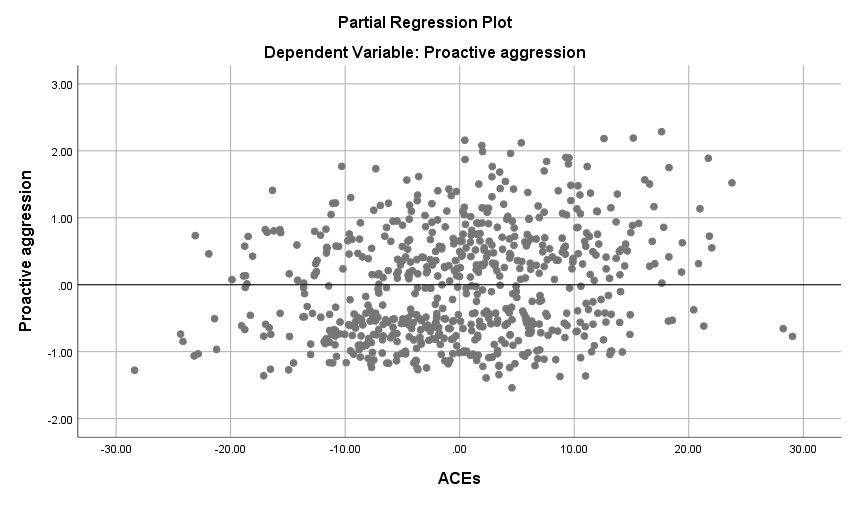 |  |

## Outcome Variable: Impulsivity Traits

| 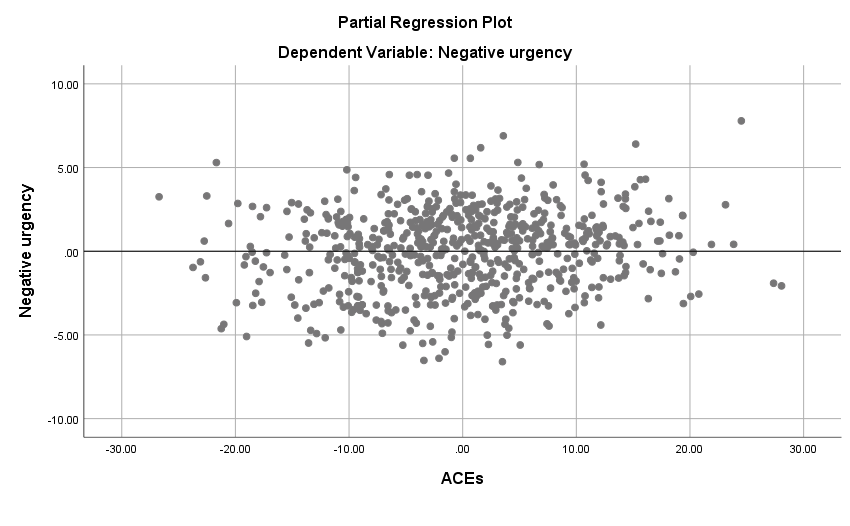 | *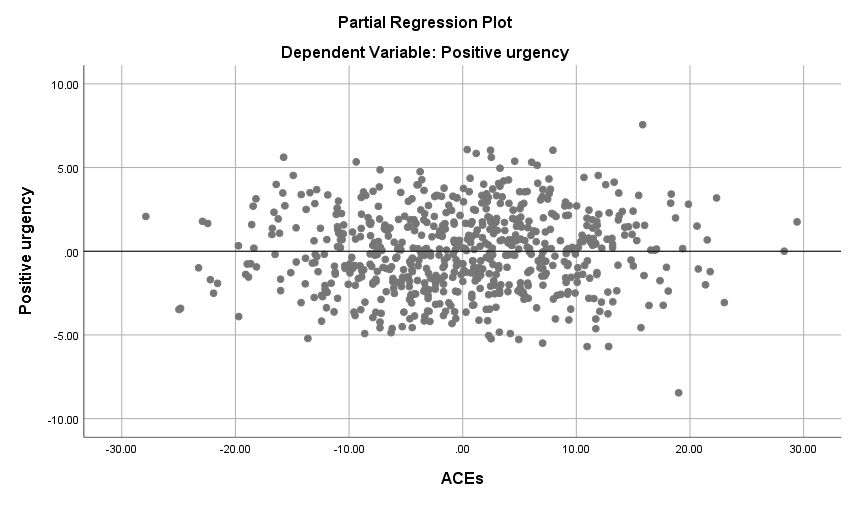* |
| --- | --- |
| *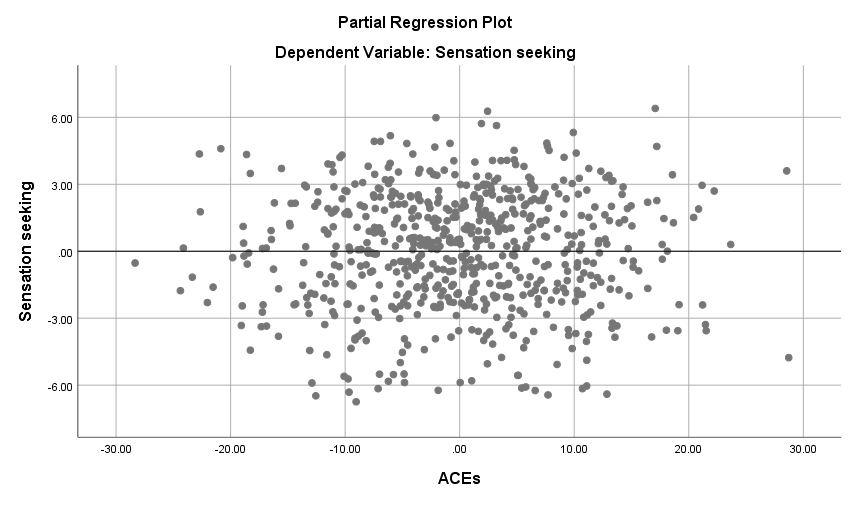* | *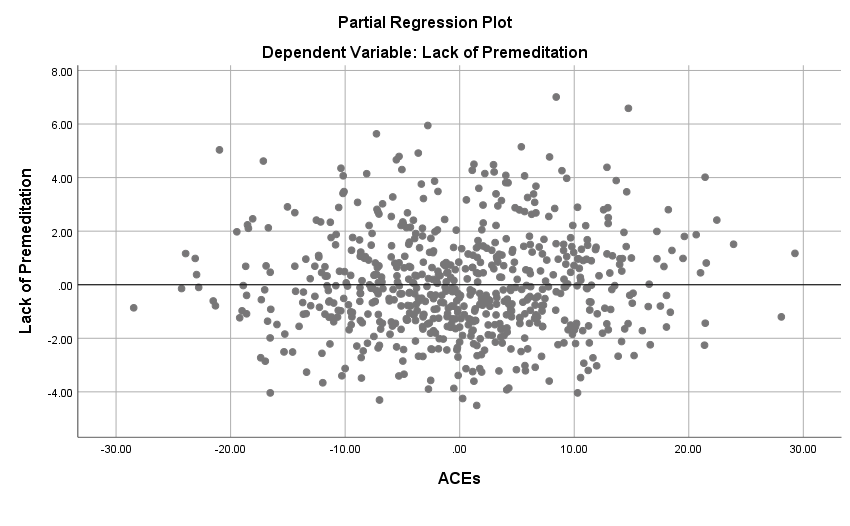* |
| *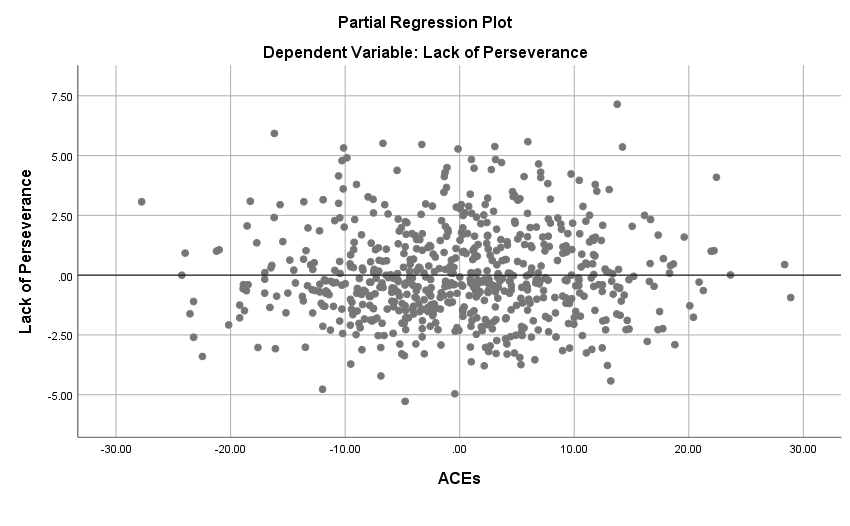* |  |

# Analysis Code

## Mediation Model with Reactive Aggression

process y= RA /x=ACE /m=NU PU PRE PER SS/cov=AGE GEN ETHN EDU/center=2/conf = 95/boot = 5000/effsize =1/total =1/stand =1/seed=31216/model=4.

## Mediation Model with Proactive Aggression

process y= PA /x=ACE /m=NU PU PRE PER SS/cov=AGE GEN ETHN EDU/center=2/conf = 95/boot = 5000/effsize =1/total =1/stand =1/seed=31216/model=4.

## Moderated Mediation Model with Reactive Aggression

process y= RA /x=ACE /m=NU PU PRE PER SS/cov=AGE GEN ETHN EDU/w=SPORT/center=2/conf = 95/boot = 5000/decimals=F10.3/moments = 1/ contrast = 1/ modelbt = 1/seed=31216/plot = 1/model=59.

## Moderated Mediation Model with Proactive Aggression

process y= PA /x=ACE /m=NU PU PRE PER SS/cov=AGE GEN ETHN EDU/w=SPORT/center=2/conf = 95/boot = 5000/decimals=F10.3/moments = 1/ contrast = 1/ modelbt = 1/seed=31216/plot = 1/model=59.

RA = Reactive aggression

PA = Proactive aggression

NU = Negative urgency

PU = Positive urgency

PRE = Lack of premeditation

PER = Lack of perseverance

AGE = Age

GEN = Gender

ETHN = Ethnicity

EDU = Education

SPORT = Sports participation
